# Retinal Involvement in COVID-19: Results From a Prospective Retina Screening Program in the Acute and Convalescent Phase

**DOI:** 10.3389/fmed.2021.681942

**Published:** 2021-06-24

**Authors:** Reema Bansal, Ashish Markan, Nitin Gautam, Rashmi Ranjan Guru, P. V. M. Lakshmi, Deeksha Katoch, Aniruddha Agarwal, Mini P. Singh, Vikas Suri, Ritin Mohindra, Neeru Sahni, Ashish Bhalla, Pankaj Malhotra, Vishali Gupta, G. D. Puri

**Affiliations:** ^1^Advanced Eye Centre, Post Graduate Institute of Medical Education and Research, Chandigarh, India; ^2^Department of Hospital Administration, Post Graduate Institute of Medical Education and Research, Chandigarh, India; ^3^Department of Community Medicine, School of Public Health, Post Graduate Institute of Medical Education and Research, Chandigarh, India; ^4^Department of Virology, Post Graduate Institute of Medical Education and Research, Chandigarh, India; ^5^Department of Internal Medicine, Post Graduate Institute of Medical Education and Research, Chandigarh, India; ^6^Department of Anesthesia and Intensive Care, Post Graduate Institute of Medical Education and Research, Chandigarh, India

**Keywords:** acute, convalescent, coronavirus disease 2019, cotton wool spots, retinopathy, retina, SARS-CoV-2, screening

## Abstract

**Objective:** To detect retinal involvement in coronavirus disease 2019 (COVID-19) patients in acute and convalescent phase by their fundus screening.

**Methods:** In a prospective, cross-sectional, observational study (July–November 2020), 235 patients (142 acute and 93 convalescent phase) underwent fundus screening in a tertiary care center in North India. For convalescent phase, “hospitalized” patients (73) were screened at least 2 weeks after hospital discharge, and “home-isolated” patients (20) were screened 17 days after symptom onset/COVID-19 testing.

**Results:** None in acute phase showed any retinal lesion that could be attributed exclusively to COVID-19. Five patients (5.38%) in convalescent phase had cotton wool spots (CWSs) with/without retinal hemorrhage, with no other retinal finding, and no visual symptoms, seen at a median of 30 days from COVID-19 diagnosis.

**Conclusions:** CWSs (and retinal hemorrhages) were an incidental finding in COVID-19, detected only in the convalescent phase. These patients were much older (median age = 69 years) than the average age of our sample and had systemic comorbidities (diabetes mellitus, hypertension, etc.). We propose the term “COVID-19 retinopathy” to denote the presence of CWSs at the posterior pole, occasionally associated with intraretinal hemorrhages, in the absence of ocular inflammation in patients with a history of COVID-19 disease.

## Introduction

Ocular involvement in coronavirus disease 2019 (COVID-19) is low and manifests mainly as conjunctivitis, in the form of conjunctival hyperemia, chemosis, increased secretions, and/or epiphora (1–3). Signs of blepharitis (crusted eyelashes, meibomian orifice alterations, lid margin hyperemia) and eye burning have also been reported (4). Dry eyes were reported in 72% of patients evaluated for neurotropism of the virus in the head–neck region (5). Retinal involvement in humans with active COVID-19 infection is uncommon. The first report of retinal findings associated with COVID-19 infection in humans described subtle retinopathy features (6). All patients were asymptomatic for ocular signs and symptoms and had normal visual acuity and pupillary reflexes. These findings, however, were strongly questioned by others (7). Subsequently, retinal microangiopathy as evidenced by cotton wool spots (CWSs) was reported in patients with previous COVID-19 infection (8). More recently, acute vascular lesions (CWSs, retinal hemorrhages, sectoral retinal infarct, etc.) were described in hospitalized, severe COVID-19 patients, with possible SARS-CoV-2 relation (9). It is not known if the retinal findings occur more frequently than reported so far, because of the asymptomatic nature of these findings. As COVID-19 has multisystem involvement, it is important not to overlook any findings in any organ, to minimize the morbidities associated with the pandemic.

Hence, we conducted a prospective study to detect retinal involvement in the acute phase of COVID-19 infection in hospitalized patients, and in convalescent-phase in patients with post-COVID infection by their fundus screening, and to establish their possible association with systemic comorbidities or COVID-19–related systemic complications.

## Methods

This was a single-center, prospective, cross-sectional, observational study of COVID-19 patients in a tertiary care referral institute in North India. The study adhered to the principles of the Declaration of Helsinki, and Institute Ethics Committee approval was obtained. Informed consent was obtained from each patient or legal representative, whenever applicable. Patients were subjected to binocular indirect ophthalmoscopy for fundus screening by an ophthalmologist. Mydriasis was achieved with tropicamide (0.8%) and phenylephrine (5%) eye drops.

Hospitalized patients with acute infection were screened in the COVID Care Center of the institute. For patients in convalescent phase, we followed the Ministry of Health and Family Welfare, Government of India, guidelines for the management of COVID-19 dated August 5, 2020^1^. The “hospitalized” patients were screened at least 2 weeks after hospital discharge, and the “home-isolated” patients were screened 17 days after symptom onset/COVID-19 testing. They were contacted by teleconsultation and requested to undergo fundus screening. Those who agreed to participate in the study were given an appointment, for the day convenient to them, to visit the department of ophthalmology. They were subjected to binocular indirect ophthalmoscopy for dilated fundus screening, after informed consent. Patients with incidentally detected fundus findings were additionally subjected to conventional fundus imaging [color/red-free fundus photography, spectral-domain optical coherence tomography (SD-OCT), and OCT angiography (OCTA)] for documentation of fundus lesions to obtain good-quality images of central and peripheral retina.

### Statistical Analysis

Statistical analyses were done using SPSS software version 17 (SPSS Inc., Chicago, IL, USA) for Windows at a 5% level of significance. Descriptive statistics included data such as mean, standard deviation, median, and range for quantitative variables.

## Results

A total of 142 patients during the acute phase and 93 patients during the convalescent phase of COVID-19 were screened for retinal examination.

The mean age of 142 patients (acute phase) was 48.16 ± 15.7 years (median = 50, range = 18–80 years). There were 84 male and 58 female patients, examined 0–31 days within the onset of COVID-19 (mean = 8.1 ± 6.64 days, median = 6 days). As per the disease classification of “asymptomatic,” “mild,” “moderate,” “severe,” and “critically ill,” (9) there were 69 mild, 29 moderate, and 44 severe disease patients screened. None of the eyes showed any retinal lesion that could be attributed exclusively to COVID-19. Retinal findings with known systemic or ocular associations were present in 27 patients (19%) and included diabetic retinopathy (8), hypertensive retinopathy (2), optic disc edema (2) [idiopathic intracranial hypertension (1), and acute pancreatitis (1)], optic disc pallor (4), optic disc cupping (2), drusen (2), chorioretinal scar (2), peripapillary atrophy (2), choroidal nevus (1), Purtscher retinopathy (1), and leukemic retinopathy (1).

Ninety-three patients were examined in convalescent phase at a mean of 37.3 ± 14.8 days (median = 30 days, range 16–86 days) from the day of diagnosis of COVID-19. The mean age was 41.09 ± 15.84 years (range = 5–74 years). There were 57 male and 36 female patients. These included 73 hospitalized (mean age = 43.09 ± 15.03 years, range = 5–74 years) and 20 home-isolated (mean age = 33.75 ± 16.95 years, range = 9–72 years) patients. Thirty-six patients were asymptomatic (16 hospitalized and 20 home-isolated); 33 had mild, 23 had moderate, and 1 had severe COVID-19.

**Figure 1:**
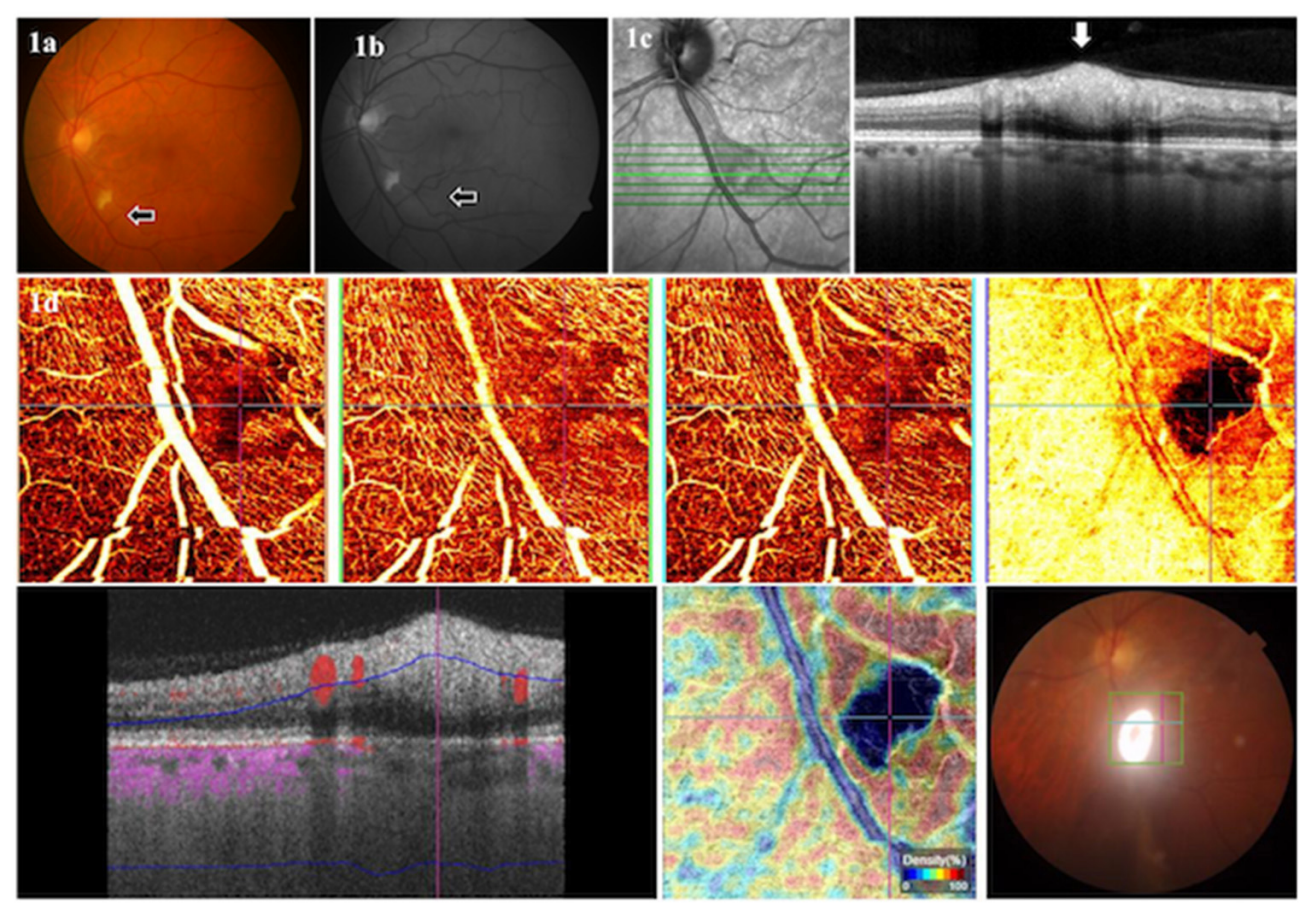
Color **(a)** and red-free **(b)** fundus photographs of a 57-year-old male patient in the convalescent phase, examined after 22 days of COVID-19 diagnosis, showing a CWSs in the left eye. The SD-OCT **(c)** showed retinal nerve fiber layer swelling (arrow) in the region of CWSs. The OCTA **(d)** showed an absence of signal (arrows) in all layers at the location of CWSs.

**Figure 2:**
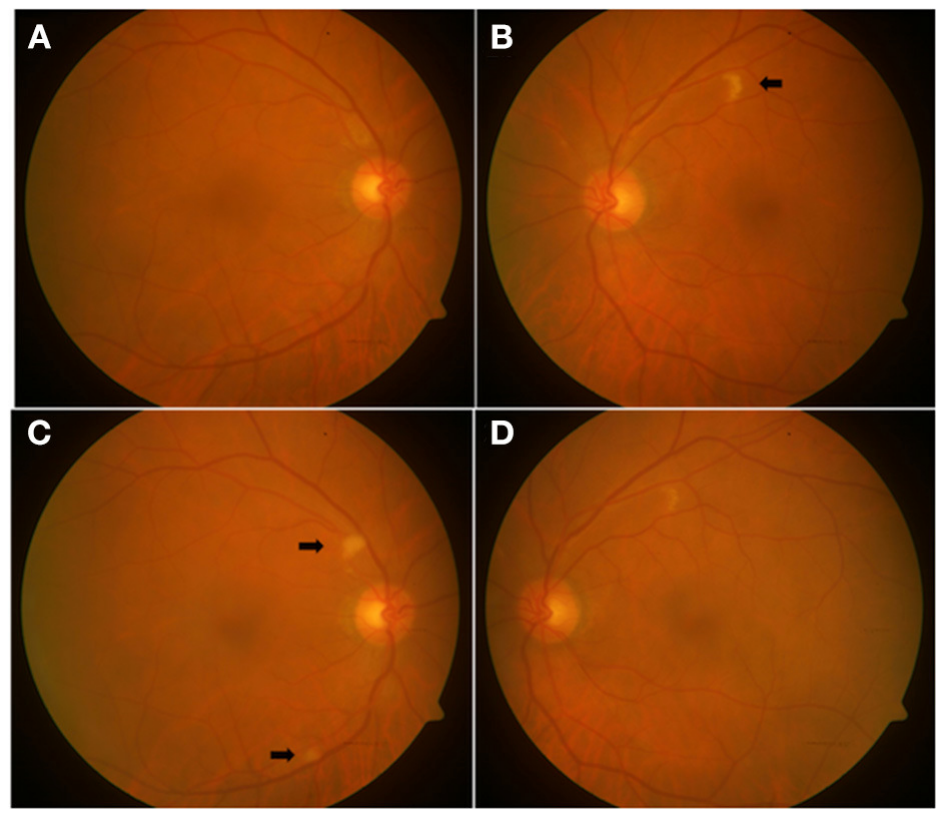
Color fundus photographs of a 73-year-old male patient in convalescent phase, examined after 44 days of COVID-19 diagnosis, showing a normal fundus of the right eye **(A)** at the first examination visit, with a CWSs in the left eye **(B)**. Two weeks later, two new CWSs (arrows) appeared in the right eye **(C)**, and the left eye showed the resolving CWSs **(D)**.

**Figure 3:**
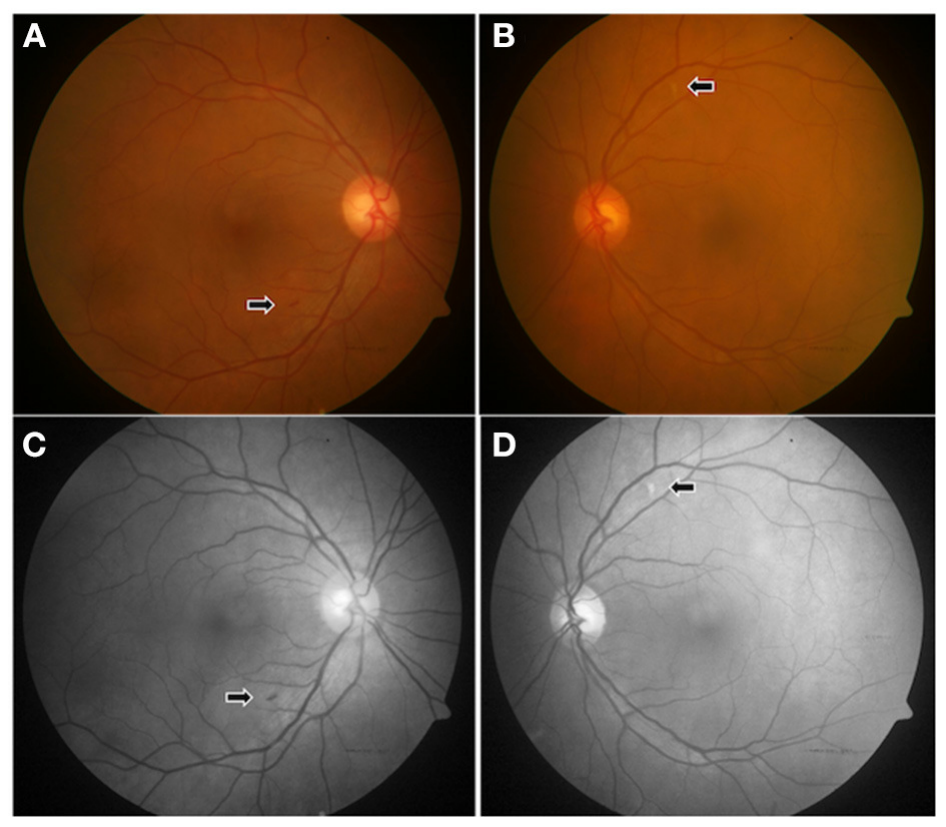
Color **(A,B)** and red-free **(C,D)** fundus photographs of a 56-year-old female patient in convalescent phase, examined after 28 days of COVID-19 diagnosis, showing a tiny flame-shaped intraretinal hemorrhage in the right eye (arrow) near the inferotemporal arcade **(A)** and a CWSs in the left eye **(B)**, along the superotemporal arcade, which is more marked (arrow) in the red-free photograph **(D)**.

Five patients (5.38%) had CWSs with/without retinal hemorrhage in the absence of any other fundus lesion (Table 1). CWSs were seen in seven eyes of five patients, and retinal hemorrhage in one eye (three had bilateral lesions and two unilateral) (Figures 1–3). The systemic comorbidities present in these patients included hypertension, diabetes mellitus, iron deficiency anemia, coronary artery disease, severe anemia, and chronic kidney disease. None had background features of diabetic, hypertensive, or anemic retinopathy. They underwent fundus screening at a mean of 30 ± 8.72 days (median = 30 days, range = 22–44 days) from the day of COVID-19 diagnosis. The mean age of these five patients was 65.4 ± 8.26 years (median = 69 years, range = 56–73 years), as compared to those (88 patients) who did not have these findings (mean age = 39.72 ± 15.1 years, median = 39.5 years, range = 5–73 years).

**Table 1:** Details of COVID-19–related retinal findings.

**Case no**.	**Sex**	**Systemic comorbidity**	**COVID-19 severity**	**Right eye**	**Left eye**	**Laterality of fundus findings**	**Days of fundus examination from diagnosis**
1	M	DM + HT	Moderate	CWS	CWS	Bilateral	44
2	M	CAD (post-CABG) + iron deficiency anemia	Mild (ILI)	CWS	CWS	Bilateral	24
3	M	DM + HT	Moderate (SARI illness)	—	CWS	Unilateral	22
4	F	Type 2 DM (recently detected)	Moderate (SARI illness)	RH	CWS (two)	Bilateral	28
5	M	DM + CAD + HT + CKD + severe anemia	Moderate (ARDS)	CWS	—	Unilateral	32

*M, male; F, female; DM, diabetes mellitus; HT, hypertension; CAD, coronary artery disease; CKD, chronic kidney disease; CWS, cotton wool spot; RH, retinal hemorrhage*.

In a 73-year-old male patient (case 1), the right eye fundus appeared normal at the first examination visit (examined after 44 days of COVID-19 diagnosis), with CWSs in the left eye. Two weeks later, two CWSs appeared in the right eye, and the left eye showed the resolving CWSs (Figure 2).

Retinal findings due to known etiologies were seen in 14 patients (15.05%), of which nine had known systemic associations [diabetic retinopathy (7), leukemic retinopathy (1), and papilledema due to idiopathic intracranial hypertension (1)], and five patients had ocular diseases [retinal myelinated nerve fibers (1), drusen (2), pigment epithelium detachment (1), and a pigmented chorioretinal scar (1)].

## Discussion

Contradictory reports of retinal findings are emerging in COVID-19, both in acute and convalescent phases (6– 12)^2^.

In a bedside eye screening of 43 severe COVID-19 pneumonia patients (mean age = 70 years) in Rome, Italy (11), Pirraglia et al. found unilateral chorioretinitis of opportunistic origin in one patient. None had retinal involvement due to SARS-CoV-2. In a large series of COVID-19 patients from Iran, diabetic retinopathy (6.3%) was the only retinal finding (12). Normal fundus findings (with no CWSs or retinal hemorrhages) were reported by Guemes-Villahoz et al. (10) in a cohort of 80 COVID-19 patients in Spain, seen within 28 to 32 days of COVID-19 diagnosis. Likewise, we did not detect any retinal finding in 142 patients, which could be exclusively related to COVID-19, in the acute phase.

However, only one study (by Pereira et al.) has reported retinal findings in hospitalized patients (Brazil) with severe COVID-19 (9), Ten patients, seen at a median of 11.5 days from COVID-19 diagnosis (range = 0–55 days), had one or more of the following: CWSs, retinal sectoral pallor, flame-shaped hemorrhages, macular scar, and choroidal nevus. Considering the high rate of thrombotic complications in critically ill COVID-19 patients in the intensive care unit (ICU) (13), the authors attributed the CWSs and sectoral retinal pallor to a thromboembolic phenomenon causing terminal retinal arteriole or branch retinal artery occlusion, respectively. Their study, however, included a small number of patients and lacked any more retinal imaging such as OCT or OCTA.

In the convalescent phase of COVID-19, five (of 93) patients had retinal findings in our series, with CWSs (seven eyes) and retinal hemorrhage (one eye). None had visual symptoms. They were much older (median = 69 years, range = 56–73 years) than the ones (88 patients) without these findings (median = 39.5 years, range = 5–73 years).

A higher prevalence [six of 27 patients (22%)] of retinal findings (cotton wool exudates) in COVID-19 convalescent patients was reported in Spain (8). The authors proposed retinal microangiopathy as an *in vivo* biomarker of systemic acute vascular disease and attributed these changes in COVID-19 to the expression of angiotensin-converting enzyme 2 (ACE2) receptor in the retina.

Marinho et al. reported subtle, hyperreflective lesions involving the papillomacular bundle in 12 patients (age range = 25–69 years) with COVID-19, without severe systemic disease (6). These lesions were seen only on OCT (localized to the ganglion cell and inner plexiform layers). The CWSs and hemorrhages were clinically visible in four of the 12 patients. Only two of their patients required hospitalization. In contrast, all our patients showing retinal findings were hospitalized [for mild (one patient) or moderate (four patients) COVID-19]. None were symptomatic for any visual complaints. They associated the involvement of ganglion cell layer and inner plexiform layers with central nervous system manifestations described in animal studies and COVID-19–related neurological events (14, 15). Vavvas et al., however, interpreted these lesions differently and proposed that the subtle lesion could be a normal finding, representing normal retinal vessels or retinal nerve fiber layer myelination, and was unlikely to be a pathological lesion (7).

Our patients with CWSs were much older (median age = 69 years) than other studies [56 years in Landecho et al.'s study (8), between 25 and 69 years in Marinho et al.'s (6) study]. We documented CWSs between 22 and 44 days from the day of COVID-19 diagnosis. However, as this depended largely on the patient's willingness and convenience to visit the hospital for fundus screening, the CWSs may have appeared earlier. This interval was 11–33 days after symptom onset in Marinho et al.'s study (6) and was 0 to 55 days in Pereira et al.'s study (9) and had a mean of 43 days in Landecho et al.'s study (8).

Invernizzi et al. observed dilatation of retinal arteries and veins [in 15 (27.7%)] that were not always detectable by clinical examination, and tortuous vessels [seven (12.9%) patients] by screening 54 severe/non-severe COVID-19 patients (excluding ICU patients and those with diabetic retinopathy), within 30 days after COVID-19 symptom onset. They also reported retinal hemorrhages [five (9.25%) patients], and CWSs [four (7.4%)] (10). The retinal vein dilatation was significantly related to the disease severity and decreased with time.

The retinal findings of CWSs and microhemorrhages indicate acute vascular events and retinal ischemia. Similar findings have been reported as a sign of retinal injury and as a manifestation of thrombotic microangiopathy (TMA) (16), although they differ in terms of significant visual loss in the latter. TMA of retinal vessels may be more common than is expected from the literature. Coagulopathy in COVID-19 has been widely reported (17, 18). Jhaveri et al. reported the first case of systemic TMA in a COVID-19 patient and demonstrated widespread microthrombi and diffuse cortical necrosis in kidney biopsy (19). Whether retinal microangiopathy is secondary to or associated with the systemic TMA seen in COVID-19 is not known.

All our patients with retinal findings had one or more systemic comorbidities, such as diabetes mellitus, hypertension, coronary artery disease, anemia, etc. None, however, had any background retinopathy changes. A possibility of enhanced damage by the virus in the retinal microvasculature in the presence of systemic comorbidities has been considered (10). An increased susceptibility of diabetic individuals to SARS-CoV-2 invasion is facilitated by CD147 (basigin), a transmembrane glycoprotein, which is also (like ACE2) expressed in human retinal cells (20–23). Raony and Saggioro de Figueiredo suggested a possible role of CD147 and cytokine storm in precipitating the retinal lesions of COVID-19 in diabetic patients (24). However, Guemes-Villahoz et al. questioned this theory, as they reported normal fundus in 80 COVID-19 patients with diabetes ^2^.

The association of COVID-19 with the vision-threatening ocular disease is emerging now, with retinal vein occlusion (25) and central retinal artery occlusion (26), possibly suggesting mechanisms of immune-complex deposition and hypercoagulability, respectively.

A study of brain autopsies from 43 patients of COVID-19 revealed mild neuropathological changes, including fresh ischemic lesions, astrogliosis, microglial activation, and cytotoxic T-lymphocyte infiltration, demonstrating neuroimmune activation involving adaptive and innate immune systems (27). A similar immune-mediated mechanism may be occurring in the retinal tissue, considering the mild nature of retinal findings, with ganglion cell and plexiform layer involvement (6). Further, in the absence of any necrotizing lesions, direct viral infection of the retina by SARS-CoV-2 is unlikely.

Gathering all the information from our study and rest of the screening studies, the clinical findings common to all these patients include an asymptomatic nature of the retinal findings and presence of subtle retinopathy in the form of CWSs with/without retinal hemorrhages, without any intraocular inflammation, particularly in the convalescent phase. However, as we did not find such lesions during the acute phase of the disease (in 142 patients) in India, similar to other studies from Italy (11), Spain^2^, and Iran (12), but unlike Pereira et al., who described these findings in acute disease (18 patients) in Brazil (9), we are unable to comment whether geographical or racial variations can influence this phenomenon.

The CWSs represent only the end-organ tissue damage. The CWSs may be secondary to an enhanced damage by systemic comorbidities, rather than direct viral damage. Herein, we propose the term “COVID-19 retinopathy” to denote the presence of CWSs at the posterior pole, which may occasionally be associated with intraretinal hemorrhages, in the absence of any signs of ocular inflammation in patients with a history of COVID-19.

As is evident from case 1 (Figure 2 and Table 1), these CWSs may continue to evolve in the same patient. It is akin to human immunodeficiency virus (HIV) retinopathy in being asymptomatic and a benign abnormality, with an uncertain etiology. However, while the HIV microvasculopathy has well-known associations with low CD4 counts and laboratory evidence of immune deficiency, we cannot, at this stage, comment upon the association of COVID-19 retinopathy with laboratory profile. So far, the evidence of COVID-19–related CWSs in the literature is limited to only patients with “symptomatic” COVID-19 (fever/dyspnea/asthenia/anosmia/bilateral pneumonia, etc.). Our patients with these findings were also hospitalized for systemic symptoms. Ours is the first study to include “systemically asymptomatic” COVID-19 patients (home-isolated) for fundus screening (in convalescent phase), none of whom had these retinal findings. Hence, although we believe that COVID-19 retinopathy occurs in patients who have been “ill,” it is pre-mature to ignore its association with “asymptomatic COVID-19,” unlike HIV microvasculopathy, which may be associated with asymptomatic HIV infection (28).

Our study has several strengths and limitations. Following the study by Pereira et al. (9), this is the only study of fundus screening in hospitalized, laboratory-confirmed (by RT-PCR) COVID-19 patients during acute illness. The current study has the largest number of patients, both in acute (142) and convalescent (93) phases. Our cohort reflects the retinal status in all disease states (asymptomatic, mild, moderate, severe) of COVID-19.

The limitations of our study are those of a typical cross-sectional study. We do not have any information on the retinal status before COVID-19 infection. Because of the various logistic issues related to COVID-19 and the pandemic, we could not enroll the same patients for screening in acute and convalescent phases. Hence, how the CWSs evolved in convalescent phase cannot be ascertained. We have not analyzed the biochemical profile of the patients screened.

The presence or absence of fundus involvement must be addressed in future surveys from other centers, particularly in severe disease. We believe that this screening, rather than being a prophylactic measure, would eliminate uncertainties about retinal involvement in COVID-19 during active as well as convalescent disease. However, the reason for relative retinal sparing in a disease so well-known to have an immune-mediated cytokine storm remains to be explored. As the CWSs indicate retinal infarcts, they may represent the earliest manifestation of subclinical systemic damage elsewhere in the body.

## Data Availability Statement

The raw data supporting the conclusions of this article will be made available by the authors, without undue reservation.

## Ethics Statement

The studies involving human participants were reviewed and approved by Institute Ethics Committee, PGIMER, Chandigarh. The patients/participants provided their written informed consent to participate in this study.

## Author Contributions

GP conceptualized and RB designed the study. The residents/fellows of the department of Ophthalmology, Advanced Eye Center, PGIMER, posted in COVID-19 duty, performed binocular indirect ophthalmoscopy and patient data collection of COVID-19 patients in the acute phase. RG and PL provided the list of post-COVID-19 patients in convalescent-phase (hospitalized, and home-isolated) for teleconsultation. AM tele-consulted and performed fundus screening of patients in convalescent-phase in the department of ophthalmology, along with data collection. NS, RM, and VS provided patient care as members of the COVID-19 team, led by PM, AB, and GP. RB led the data collection, performed the analysis, and prepared results. MS supervised the laboratory confirmation of all COVID-19 patients. DK supervised the MII-RetCam fundus imaging in COVID-19 ward. NG performed the fundus imaging of convalescent-phase patients. VG oversaw the research. RB wrote the first draft of the manuscript, with inputs from DK, AA, VG, AM, NG, RG, PL, MS, VS, RM, NS, AB, PM, and GP. RB and AM verify the underlying data. All authors contributed to the article and approved the submitted version.

## Conflict of Interest

The authors declare that the research was conducted in the absence of any commercial or financial relationships that could be construed as a potential conflict of interest.

## Footnotes

^1^https://www.mohfw.gov.in/pdf/ReviseddischargePolicyforCOVID19.pdf

^2^http://www.mohfw.gov.in/pdf/RevisedNationalClinicalManagementGuidelineforCOVID1931032020.pdf
